# Thermodynamic controls of the Atlantic Niño

**DOI:** 10.1038/ncomms9895

**Published:** 2015-11-26

**Authors:** Hyacinth C. Nnamchi, Jianping Li, Fred Kucharski, In-Sik Kang, Noel S. Keenlyside, Ping Chang, Riccardo Farneti

**Affiliations:** 1 Department of Geography, University of Nigeria, Nsukka 410001, Nigeria; 2 College of Global Change and Earth System Science, Beijing Normal University, Beijing 100875, China; 3 Joint Center for Global Change Studies, Beijing 100875, China; 4 Earth System Physics Section, Abdus Salam International Centre for Theoretical Physics, Strada Costiera, 11, Trieste 34151, Italy; 5 School of Earth and Environmental Sciences, Seoul National University, Seoul 151-742, South Korea; 6 Geophysical Institute, University of Bergen and Bjerknes Centre for Climate Research, Allégaten 70, 5007 Bergen, Norway; 7 Department of Oceanography, Texas A&M University, College Station, Texas 77843-3160, USA; 8 Collaborative Innovation Center of Marine Science and Technology, Ocean University of China, Qingdao 266100, China

## Abstract

Prevailing theories on the equatorial Atlantic Niño are based on the dynamical interaction between atmosphere and ocean. However, dynamical coupled ocean-atmosphere models poorly simulate and predict equatorial Atlantic climate variability. Here we use multi-model numerical experiments to show that thermodynamic feedbacks excited by stochastic atmospheric perturbations can generate Atlantic Niño s.d. of ∼0.28±0.07 K, explaining ∼68±23% of the observed interannual variability. Thus, in state-of-the-art coupled models, Atlantic Niño variability strongly depends on the thermodynamic component (*R*^2^=0.92). Coupled dynamics acts to improve the characteristic Niño-like spatial structure but not necessarily the variance. Perturbations of the equatorial Atlantic trade winds (∼±1.53 m s^−1^) can drive changes in surface latent heat flux (∼±14.35 W m^−2^) and thus in surface temperature consistent with a first-order autoregressive process. By challenging the dynamical paradigm of equatorial Atlantic variability, our findings suggest that the current theories on its modelling and predictability must be revised.

## Abstract (web summary)

The nature of the El Niño-like variability in the Atlantic Ocean and its limited predictability remain unresolved. Here, via multi-model numerical experiments, the authors show that much of the variability can be explained by the interaction of stochastic atmospheric fluctuations with the ocean mixed layer.

## Graphical abstract

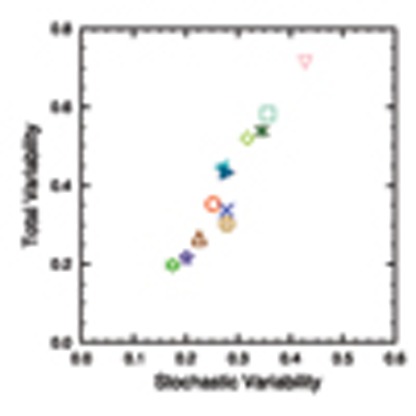

The equatorial Atlantic Ocean exhibits sporadic interannual fluctuations in zonal sea surface temperature (SST) gradients associated with westerly wind perturbations^1^,^2^,^3^,^4^. This dominant coupled ocean-atmosphere mode of behaviour is often considered the Atlantic equivalent of the Pacific El Niño and is hence referred to as the Atlantic Niño^1^,^2^. The Atlantic Niño peaks during the boreal summer (June-July-August), when intensified equatorial upwelling typically leads to the development of the cold tongue^5^. Atmospheric convection and circulation changes due to the Atlantic Niño can cause increased precipitation across the equatorial Atlantic and decreases over the Sahel^6^. Beyond the Atlantic sector, the Atlantic Niño can excite near-global scale tropical atmospheric teleconnections, causing shifts in weather regimes in parts of the Americas, Africa, the Mediterranean, India and beyond^6^,^7^,^8^,^9^. Indeed, a growing body of evidence shows that the equatorial Atlantic modulates, and could therefore enhance the predictability of, the equatorial Pacific events^2^,^10^,^11^,^12^,^13^.

These far-reaching effects call for an improved understanding of the physical processes that underlie equatorial Atlantic variability. As in the case of the Pacific El Niño, the leading theory for the equatorial Atlantic Niño is based on Bjerknes dynamical feedbacks^1^,^2^,^3^,^14^,^15^. The theory postulates that zonal wind perturbations {*u*}' over the western equatorial Atlantic can drive ocean currents and equatorial baroclinic waves that redistribute upper ocean heat content, thereby modulating SST change in the eastern basin. Studies motivated by the Bjerknes theory show that the peak phase of the Atlantic Niño in June-July-August is preceded by equatorial westerly wind perturbations ({*u*}'>0) (refs 1, 2). However, compared with that of the Pacific, the Bjerknes coupling strength is of the order of 50% weaker^2^,^16^ and {*u*}' accounts for only ∼15–35% of observed equatorial Atlantic variability^17^. In addition to the Bjerknes theory, recent studies propose that intrinsic dynamics of equatorial deep zonal jets^18^ and meridional advection of warm water from the north tropical Atlantic^17^ contribute to Atlantic Niño variability. To date, all existing theories on the Atlantic Niño are largely inspired by dynamical thinking.

But how essential is dynamical coupling between the ocean and atmosphere to equatorial Atlantic variability? We explore this question by comparing simulations of two sets of experiments using 12 different climate models^19^ (see Methods). The first set of experiments is based on state-of-the-art, fully coupled general circulation models (CGCMs) (full-CGCMs, hereafter), and in the second set, dynamical feedbacks are disabled by thermodynamically coupling the atmosphere to a 50-m deep slab of motionless ocean (slab-CGCMs). We show that thermodynamic mechanisms play dominant roles in both sets of experiments, accounting for 68±23% of observed equatorial Atlantic interannual SST variability in the slab-CGCMs, through the wind-evaporation-SST feedbacks^20^. The impact of coupling to a dynamical ocean model is to amplify the variability and the Niño-like spatial structure. We conclude that, driven largely by stochastic atmospheric forced heat and moisture fluxes, the Atlantic Niño is not different from a first-order autoregressive (AR(1)) process.

## Results

### Equatorial winds preconditioning of the Atlantic Niño

We first examine the relationship between the Atlantic Niño SST anomaly {*SST*}' during its peak season of boreal summer and the preceding season near surface {*u*}' over the western equatorial Atlantic Ocean as a measure of Bjerknes feedback in the full-CGCMs. We then introduce the slab-CGCMs and describe the contributions from thermodynamic feedbacks only.

**Figure 1:**
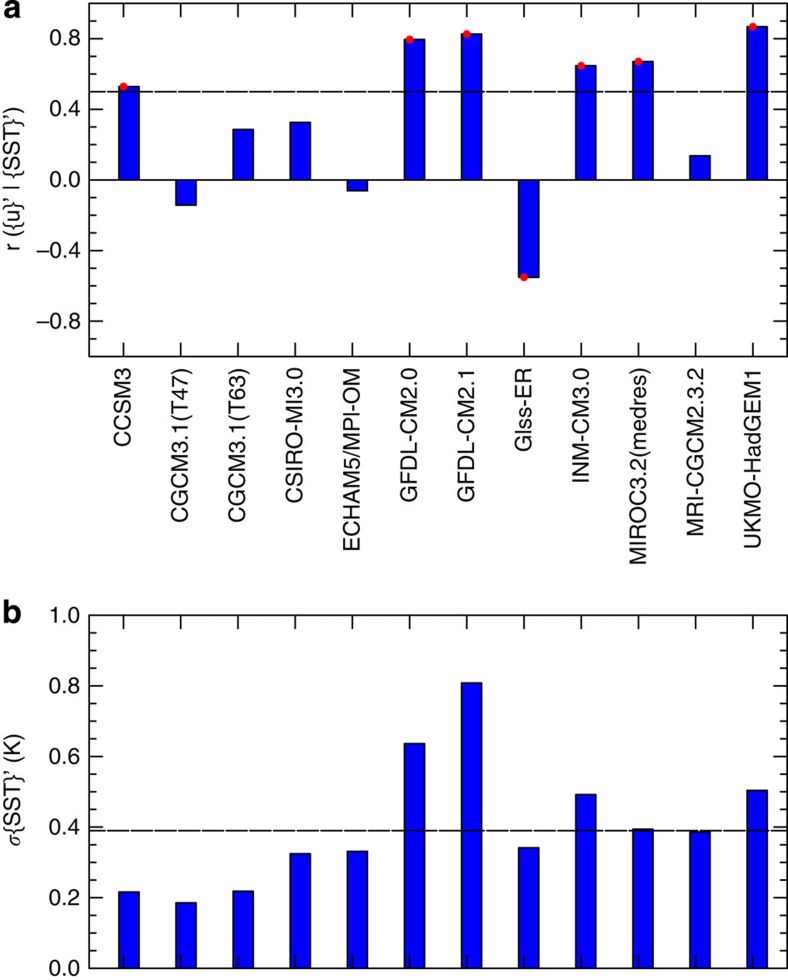
Effect of the equatorial Atlantic westerly wind perturbations on Atlantic Niño variability in fully coupled models. (**a**) Correlation between the {*u*}' averaged over the western equatorial Atlantic Ocean (2°N–2°S, 10°W–40°W) during March-April-May (MAM) and {*SST*}' averaged over 3°N–3°S, 5°W–15°W during June-July-August (JJA), following the approach of ref. 17. The {*u*}' at 925-hPa was analysed for models (CCSM3, CSIRO-Mk3.0 and ECHAM5/MPI-OM) for which the surface winds were not available from the Coupled Models Intercomparison Project, phase 3 archive. Red dots denote statistical significance at *P*≤0.001. Horizontal black line denote mean observational results^17^. (**b**) Standard deviations of the Atlantic Niño SST index (*σ*{*SST*}') in JJA. Horizontal black line denote observational data sets estimates. In each panel, the abscissa shows the model names.

The relationship between {*u*}' and {*SST*}' exhibits wide variations across the full-CGCMs (Fig. 1). The {*u*}'*|*{*SST*}*'* correlation is robust (*P*≤0.001) and of the same sign as observations for only six of the 12 models. As expected, models of overly strong {*u*}'*|*{*SST*}' coupling (for example, GFDL-CM2.0, GFDL-CM2.1 and UKMO-HadGEM1) tend to overestimate equatorial Atlantic SST variability represented by the s.d. (*σ*{*SST*}*'*) of the Atlantic Niño index. However, it is rather surprising that some models (CSIRO-Mk3.0 and MRI-CGCM2.3.2) with weak {*u*}'*|*{*SST*}' coupling exhibit realistic *σ*{*SST*}'. Indeed, even models with weak (ECHAM5/MPI-OM) and statistically significant (GISS-ER) but incorrect {*u*}'*|*{*SST*}' correlation signs, can generate realistic *σ*{*SST*}' of the Atlantic Niño.

Furthermore, some numerical models (for example, CGCM3.1_T47, GISS-ER and MRI-CGCM2.3.2) that simulate the correct structure of seasonality (Supplementary Fig. 1)— usually considered a distinctive feature of the Atlantic Niño^1^,^2^—fail to predict the expected {*u*}'*|*{*SST*}' relationship. Indeed, the multi-model variance of equatorial Atlantic *σ*{*SST*}' explained by the {*u*}'*|*{*SST*}' coupling is only ∼37%, suggesting that processes other than the Bjerknes feedback may be key to equatorial Atlantic variability.

### Thermodynamic forcing of the equatorial Atlantic Niño

In reality, SST variability is caused by a combination of dynamical (three-dimensional temperature advection) and thermodynamic (surface net heat flux, in part driven by stochastic atmospheric perturbations) feedbacks on the ocean mixed layer. Indeed, recent studies have questioned the essence of ocean dynamics in generating the equatorial Pacific El Niño, concluding that thermodynamic feedbacks may be more important than previously thought^21^,^22^,^23^,^24^. However, the effect of stochastic atmospheric induced heat and moisture fluxes in the origin of the equatorial Atlantic Niño remains largely unknown and is thus examined in the present study.

**Figure 2:**
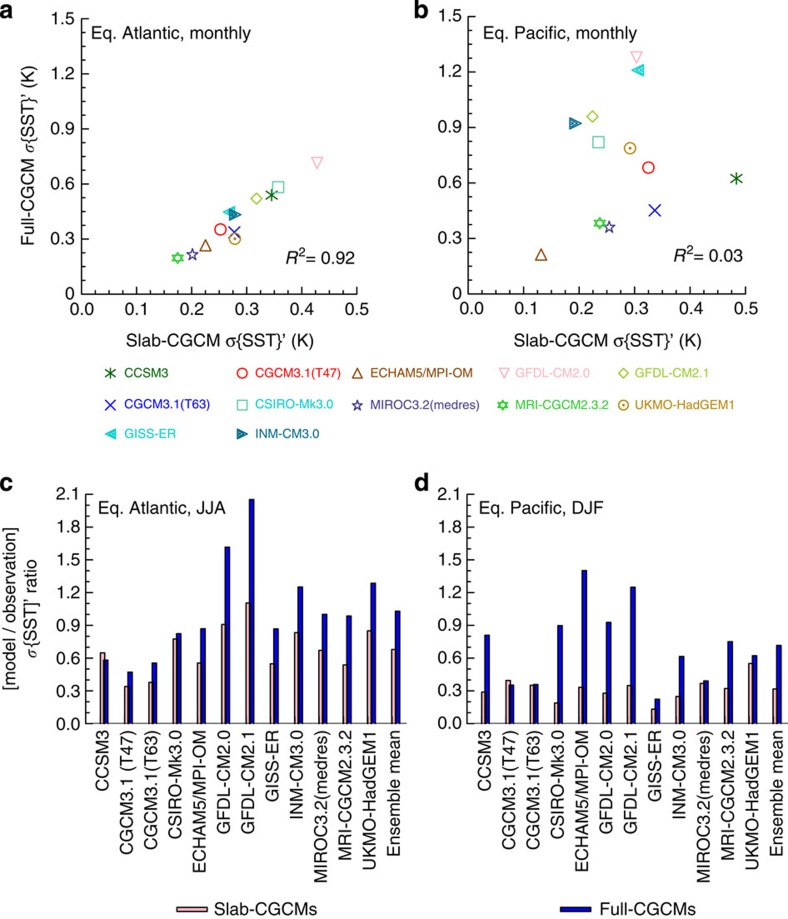
Multi-model linearity of thermodynamic feedbacks in the eastern equatorial Atlantic and Pacific oceans and modelled proportions of the observed variability. (**a**,**b**) Relationship between the monthly Atlantic Niño (3°S–3°N, 0–20°W) and Pacific Niño-3 (5°S–5°N, 90–150°W) *σ*{*SST*}' from slab-CGCMs and full-CGCMs. (**c**,**d**) Atlantic Niño and Pacific Niño-3 model/observation ratio of the *σ*{*SST*}'. Blue bars denote the ratios for full-CGCMs; light-pink, slab-CGCMs. A ratio of >1.0 denotes an overestimation of observed variability of ∼0.40 K and ∼0.95 K for the Atlantic Niño and Pacific Niño-3, respectively. Panels c and d are based on the JJA and December-January-February (DJF) seasonal means, respectively.

The monthly Atlantic Niño index of the 12 slab-CGCMs exhibits strong variability (*σ*=∼0.28±0.07 K; for the full-CGCMs, *σ*=∼0.47±0.09 K). Figure 2 shows that the *σ*{*SST*}*'* of the Atlantic Niño of the full-CGCMs (representing the total variability of the equatorial Atlantic ocean-atmosphere coupled system) is linearly dependent on the *σ*{*SST*}*'* of the slab-CGCMs with no interactive ocean dynamics. The multi-model correlation coefficient is 0.96, corresponding to 92% of the explained variance. In comparison, the equatorial Pacific variability represented by the Niño-3 index exhibits more scatter with an explained variance of only 3%. This implies that while dynamical coupling in the equatorial Atlantic acts to amplify the *σ*{*SST*}*'*, coupled dynamics in the Pacific tends to modify the SST variability.

We determined the proportions of the observed SST variability over the eastern equatorial Atlantic Ocean resulting from thermodynamic processes by computing the boreal summer ratios of the modelled *σ*{*SST*}' to recent historical observations^25^,^26^,^27^(1984–2013). The results show that 10 models have ratios of >0.50, with the 12 slab-CGCMs ensemble-mean of ∼68±23%, suggesting that thermodynamic processes dominate the equatorial Atlantic interannual SST variability (Fig. 2c). To put this result in context, similar ratios were calculated for the boreal winter peak season of the equatorial Pacific El Niño using the Niño-3 index. The corresponding ensemble-mean contribution of thermodynamic feedbacks for the Niño-3 region is considerably lower at ∼32±11% (Fig. 2d).

The full-CGCM ratios were also plotted on the same axes of Fig. 2c,d. The observed ocean mixed layer in the equatorial Atlantic exhibits marked annual cycles and is essentially shallow (<50 m) in the Atlantic Niño region (Supplementary Fig. 2). Thus, some models with deep mixed layer of generally >50 m (for example, CCSM3, CGCM3.1_T47 and CGCM3.1_T63) tend to underestimate the observed *σ*{*SST*}'. In contrast, for those models in which the thermodynamic component generate *σ*{*SST*}' close to observations (for example, GFDL-CM2.0, GFDL-CM2.1, INM-CM3.0 and UKMO-HadGEM1), coupling to ocean dynamics leads to unrealistically strong amplitudes of the Atlantic Niño *σ*{*SST*}'—given that the mixed layer depths are not much different from the observed. The ensemble-mean of the full-CGCMs reproduces the equatorial Atlantic SST temporal variability as may be expected from the consistency of the modelled mean mixed layer depths with observations.

To determine the comparative fidelity of the full-CGCM and slab-CGCM models in reproducing the observed spatial structure of the Atlantic Niño, we extracted the associated SST variability over the equatorial Atlantic Ocean (10°N–10°S, 20°E–60°W) as the leading empirical orthogonal function (Supplementary Fig. 3). This analysis essentially precludes that a model may still have an Atlantic Niño that is not the leading mode; we assume that if this occurs, it means that the model is unrealistic. The accuracy of the models is evaluated in relation to observations and the results quantified via second-order statistics^28^.

**Figure 3:**
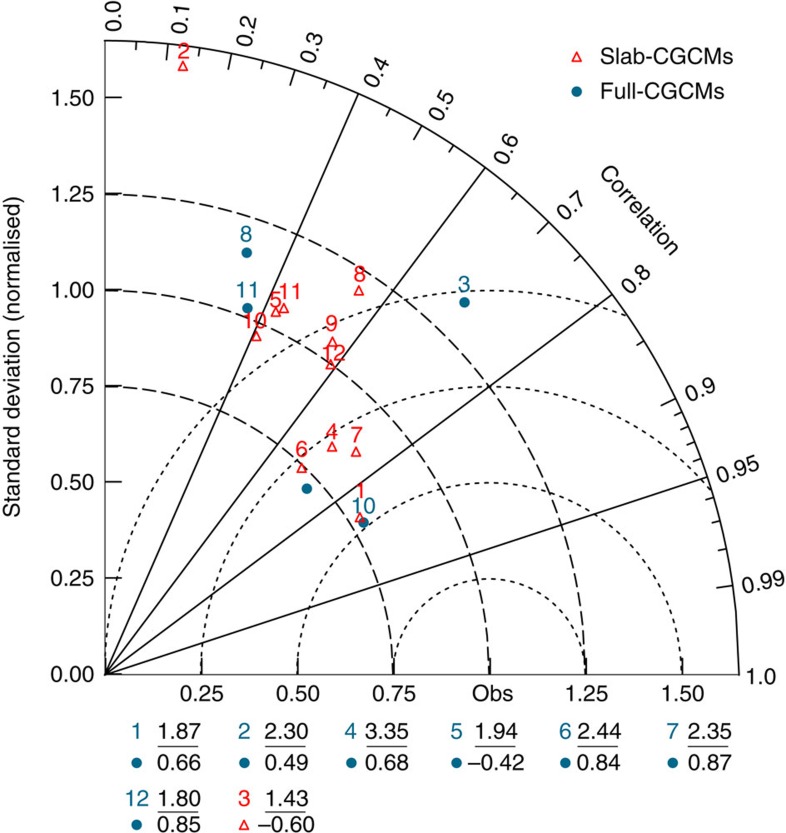
Comparative evaluation of the spatial patterns of equatorial Atlantic {*SST*}' simulated in thermodynamic and fully coupled control experiments. For each model, the leading empirical orthogonal function of the {*SST*}' is computed for the equatorial Atlantic Ocean (10°N–10°S, 20°E–60°W), and the spatial pattern determined by regressing the {*SST*}' onto the associated time series. The radial coordinate denotes the magnitude of total s.d., normalized by the observed value; the angular coordinate represents the correlation with observations^25^,^26^,^27^. Distances between ‘obs' denoting observed point and any model point is proportional to the r.m.s. model error. Outliers (defined as models with negative correlations and/or *σ*>1.65) are listed below the plot. 1-CCSM3; 2-CGCM3.1(T47); 3-CGCM3.1(T63); 4-CSIRO-Mk3.0; 5-ECHAM5/MPI-OM; 6-GFDL-CM2.0; 7-GFDL-CM2.1; 8-GISS-ER; 9-INM-CM3.0; 10-MIROC3.2(medres); 11-MRI-CGCM2.3.2; 12-UKMO-HadGEM1.

Among the six models exhibiting realistic representation of equatorial Atlantic SST variability, defined as those falling within the third arc from observations, four (CCSM3, CSIRO-Mk3.0, GFDL-CM2.0 and GFDL-CM2.1) are slab-CGCMs and only two (INM-CM3.0 and MIROC3.2_medres) are fully coupled (Fig. 3). These two full-CGCMs with a more realistic leading mode have both realistic *σ*{*SST*}' and {*u*}'*|*{*SST*}' coupling (Fig. 1).

On the other hand, those full-CGCMs with realistic *σ*{*SST*}' despite weak or incorrect {*u*}'*|*{*SST*}' correlation (for example, CSIRO-Mk3.0, ECHAM5/MPI-OM, GISS-ER and MRI-CGCM2.3.2), fail to reproduce the observed spatial structure of the equatorial Atlantic SST variability. These results suggest that while thermodynamic feedbacks can generate strong *σ*{*SST*}', the characteristic spatial structure of the Atlantic Niño could arise from coupled dynamics. Indeed, in most models (for example, CGCM3.1_T47, CGCM3.1_T63, GFDL-CM2.0, GFDL-CM2.1, INM-CM3.0, MIROC3.2_medres and UKMO-HadGEM1), coupling to ocean dynamics actually improves the spatial correlation with observations, but not the amplitudes of the SST variability. The implication is that, overall, the fully coupled models are no better than their thermodynamic counterparts in simulating the spatial structure of the Atlantic Niño.

**Figure 4:**
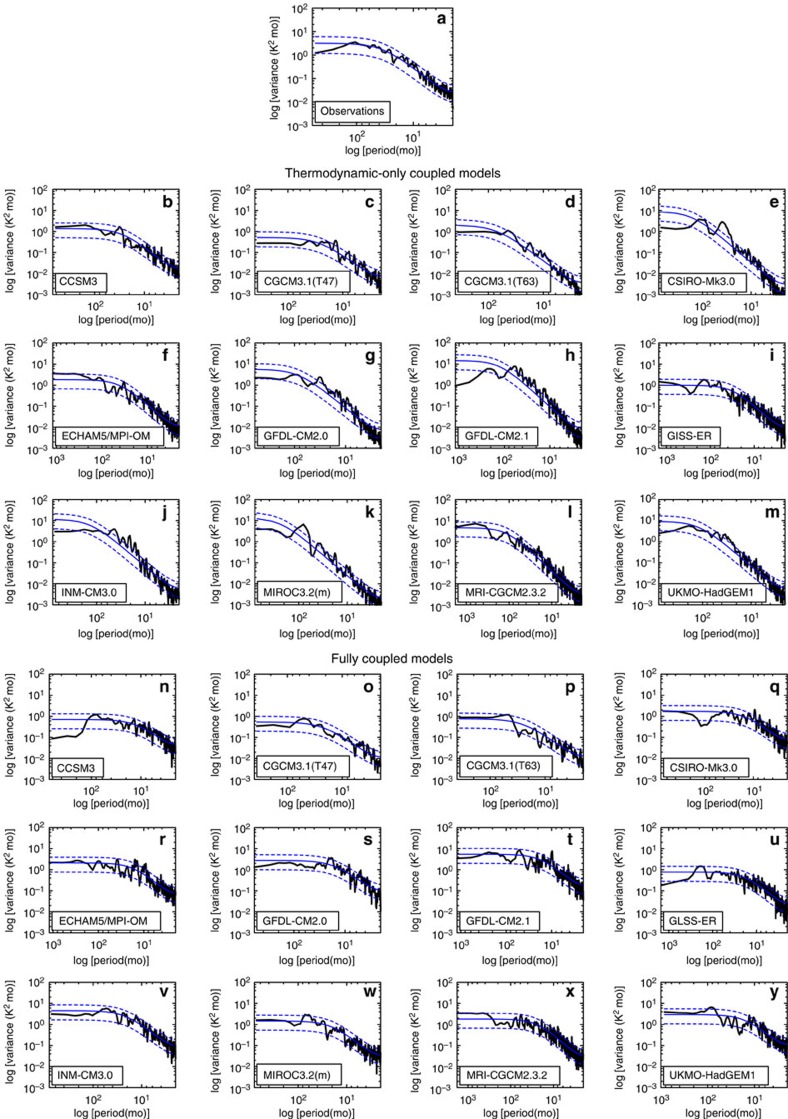
Observed and modelled spectra of Atlantic Niño. (**a**) Observed^25^ monthly spectrum of the Atlantic Niño index for 1984–2013. Panels (**b**–**m**) Atlantic Niño spectra simulated by the slab-CGCMs. (**n**–**y**), Atlantic Niño spectra simulated by the full-CGCMs. The model names are indicated on the bottom left corner of each panel. For the observations and models, the thick back curves represent the Atlantic Niño spectra; solid blue curves denote the AR(1) spectra; dashed blue curves, the 95% confidence bounds of the AR(1) spectra.

Previous studies suggest that the Atlantic Niño exhibits interannual periodicity (see ref. 18 and references therein). However, as shown in Fig. 4, the supposed interannual peaks of the observed and modelled monthly Atlantic Niño spectrum are hardly statistically different from an AR(1) process consistent with the spectrum in an earlier analysis^29^. A comparison of the modelled spectra reveals that rather than the widely discussed coupled dynamics, the AR(1) spectral properties of the equatorial Atlantic SST variability are well reproduced with thermodynamic feedbacks alone (Fig. 4b–m), as predicted by simple stochastic climate models in which the ocean mixed layer integrates random atmospheric perturbations^30^,^31^. Coupling to ocean dynamics tends to increase the interannual, but not the low-frequency, variance of the Atlantic Niño, but the spectra retain their AR(1) character (Fig. 4n–y).

### Evolution and heat budget of the Atlantic Niño

The above analysis clearly shows that thermodynamic processes are alone able to reproduce aspects of Atlantic Niño variability. Nevertheless, there remains a wide spread of model performance that is further exacerbated by coupling to a dynamical ocean model, as may be expected from larger mean biases of the full-CGCMs. Our aim is not to fully account for inter-model differences, but instead to investigate the physical processes involved. In this respect, we further analyse GFDL-CM2.0 (ref. 32) as its thermodynamic configuration captures the space-time variability of the Atlantic Niño fairly well (Figs 2, 3, 4). The fully coupled configuration of GFDL-CM2.0 simulates strong {*u*}'*|*{*SST*}'—suggesting that Bjerknes feedbacks operate in the model—in the equatorial Atlantic Ocean.

**Figure 5:**
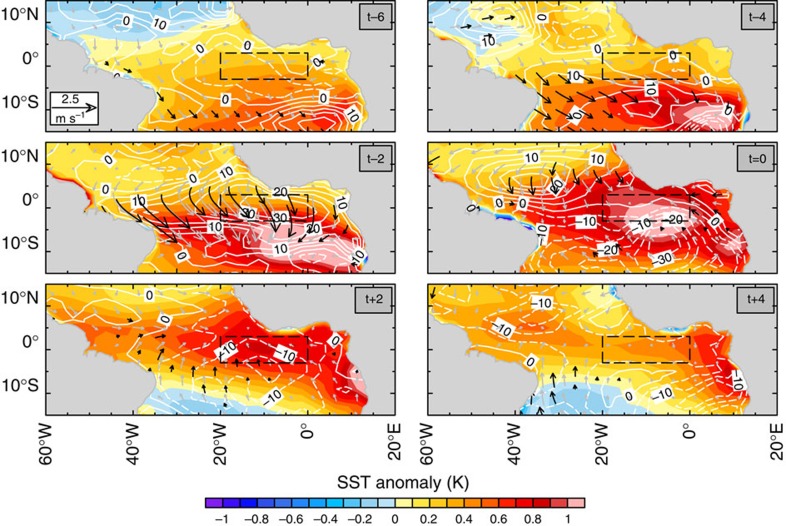
Surface net heat flux and winds associated with Atlantic Niño in the thermodynamic configuration of GFDL-CM2.0. Evolution maps of monthly anomalies of SST (colour scale: unit, K), *Q*_NET_ (contours: unit, W m^−2^) and winds (arrows: unit, m s^−1^) regressed on the Atlantic Niño index fixed at *t=*0, corresponding to July. Dashed black box indicate the Atlantic Niño domain defined by ref. 1. Thick black arrows represent statistically significant (*P*≤0.05) wind anomalies. The net heat flux is contoured at an interval of 5 W m^−2^; positive (negative) values indicate that the ocean mixed layer is gaining (loosing) heat.

As shown in Fig. 5, initial {*SST*}' forms off southeastern tropical Atlantic Ocean; that is, the Benguela Niño sector at *t-*6 leading to the peak phase of the Atlantic Niño. In subsequent months, the maximum {*SST*}' propagates north-westwards, amplifies to cover the equatorial Atlantic Ocean and then begins to decay. In the absence of ocean dynamics, the ocean mixed layer warming and cooling rates (the time derivative of temperature, ∂*T/*∂*t*) are governed entirely by net surface heat flux anomaly ({*Q*_NET_}') driven by stochastic atmospheric perturbations. Thus, at *t–*6, the {*Q*_NET_}' in the Benguela Niño area is associated with weakening of the trade winds and westerly wind perturbations ({*u*}'>0) in the equatorial region. The following months are marked by a progressive northward intensification of the westerlies and {*Q*_NET_}' that drives the {*SST*}'.

Observational studies show that net heat flux plays a significant role in the annual cycle of equatorial Atlantic Ocean SST^33^,^34^,^35^. Similarly, a modelling analysis shows that the interaction of SST with radiation and evaporation feedbacks can produce quasi-biennial equatorial Atlantic variability^36^. Some previous studies, however, suggest that interannual {*Q*_NET_}' dampens rather than drive the {*SST*}' in the region, although the explained variance is relatively low^37^,^38^,^39^. The upper ocean heat budget in the tropical Atlantic involves exceedingly complex feedback processes^4^,^17^,^18^,^36^,^40^, and outcomes may depend on specific domains analysed. Furthermore, the evolution maps indicate that the relationship between {*Q*_NET_}' and {*SST*}' actually undergoes a phase change during the life cycle of the Atlantic Niño (Fig. 5). The {*Q*_NET_}' drives {*SST*}' during the development stages. Once the peak phase is reached and the associated westerly and northerly wind anomalies decline, the relation is reversed and {*Q*_NET_}' begins to reduce {*SST*}', leading to the decay of an Atlantic Niño event. Thus, the {*Q*_NET_}' averaged in the Atlantic Niño region is +28.58 W m^−2^ at *t–*2, −4.08 W m^−2^ at *t*=0 and −16.15 W m^−2^ at *t+*3.

**Figure 6:**
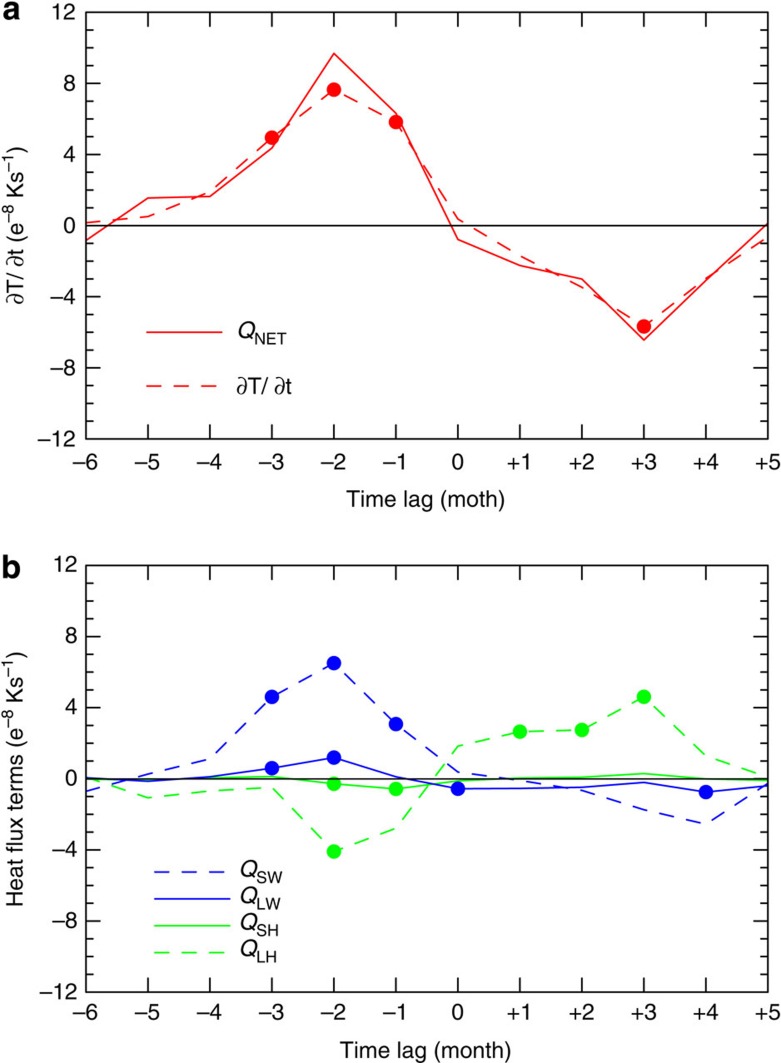
Atlantic Niño heat budget in the thermodynamic configuration of GFDL-CM2.0. (**a**) Composite evolution of anomalies of the ∂*T/*∂*t* (dashed curve) and *Q*_NET_ (solid curve). (**b**) Individual terms of *Q*_NET_: composite anomalies of *Q*_SW_ (blue, dashed), *Q*_LW_ (blue, solid), *Q*_SH_ (green, solid) and *Q*_LH_ (green, dashed). Composites are based on a +1.0*σ* of the Atlantic Niño SST index at lag *t*=0 for July. Note that *Q*_SW_ is defined positive downward, while the other fluxes are defined positive upwards. Dots denote statistical significance at *P*≤0.05.

The {*Q*_NET_}' is determined by ocean-atmosphere feedbacks resulting from four different flux components, expressed here as anomalies: shortwave radiation {*Q*_SW_}', longwave radiation {*Q*_LW_}', latent heat {*Q*_LH_}' and sensible heat {*Q*_SH_}' (see Methods). The evolution of the {∂*T/*∂*t*}' over the equatorial Atlantic Ocean during a typical Atlantic Niño year is closely reproduced by the {*Q*_NET_}' such that the residual term is quite small (Fig. 6a). Here {∂*T/*∂*t*}' is largely controlled by shortwave radiation ({*Q*_SW_}'=+15.83 W m^−2^) and by forcing from negative latent heat flux ({*Q*_LH_}'=−14.35 W m^−2^) peaking at *t–*2 during the development phase of the Atlantic Niño. The {*Q*_LW_}' and {*Q*_SH_}' appear to play comparatively minor roles. Superimposed on the climatological-mean state, negative {*Q*_LH_}' represents a suppression of evaporation due to a weakening of the trade winds, giving rise to net surface heating. Thus, the {*Q*_LH_}' and {*Q*_NET_}' tend to evolve together (Supplementary Fig. 4). Once the mature phase of Atlantic Niño is reached, {*Q*_LH_}' also appears vital to the decay of the {*SST*}'. The evolution of {*Q*_LH_}' from negative to positive suggests that as the equatorial westerly perturbations weakens, the trade winds strengthen, thereby intensifying evaporative cooling and the decay of Atlantic Niño {*SST*}'.

While these feedback mechanisms dominate in slab-CGCMs, in which ocean-atmosphere coupling is solely determined by thermodynamics, it is imperative to understand their relative contributions in a fully coupled model that also includes mixed layer temperature advection. Unlike the slab-CGCMs and earlier analysis^17^ based on some assumed constant depths, we diagnose the heat budget of 500-year integration of the fully coupled configuration of GFDL-CM2.0 by explicitly accounting for the time evolution of the ocean mixed layer depth (see Methods). The GFDL-CM2.0 partly captures the annual cycle of the equatorial Atlantic thermocline consistent with observations^41^ and reanalysis^42^ data sets, although its shoaling is somewhat delayed (Supplementary Fig. 5).

**Figure 7:**
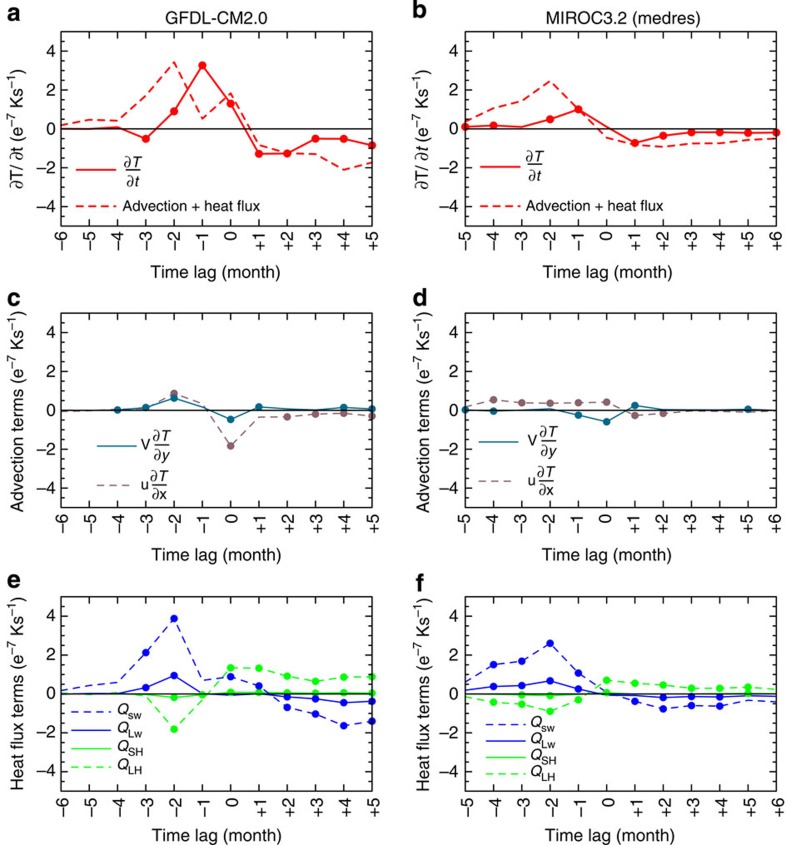
Atlantic Niño heat budget in fully coupled configurations of GFDL-CM2.0 and MIROC3.2_medres models. (**a**) Composite evolution of anomalies of ∂*T/*∂*t* (solid curve) and the sum of advection and *Q*_NET_ terms (dashed curve). (**c**) individual temperature advection terms: composite anomalies of the *u*∂*T/*∂*x* (pink, dashed) and *v*∂*T/*∂*y* (blue, solid). (**e**) Q_NET_ terms: composite anomalies of *Q*_SW_ (blue, dashed), *Q*_LW_ (blue, solid), *Q*_SH_ (green, solid) and *Q*_LH_ (green, dashed). Note that *Q*_SW_ is defined positive downward, while the other fluxes are defined positive upwards. The composites are based on a +1.0*σ* of the Atlantic Niño SST index at lag *t*=0 for July; dots denote statistical significance at *P*≤0.05. Similar plots are shown on the right panels (**b**,**d**,**f**) based on the MIROC3.2_medres for +1.0*σ* composites of the Atlantic Niño SST index at lag *t*=0 for the month of peak seasonality, June.

Figure 7a shows that the peak of the sum of temperature advection and {*Q*_NET_}' terms at *t*-2 results in the peak of {∂*T/*∂*t*}*'* after a month, this phase-lag may be attributed to the inability of the monthly time series analysed to fully resolve the heat budget in time and neglecting other terms such as vertical advection and mixing. Nevertheless, the {*Q*_NET_}' terms drive the SST change; the time average of {∂*T/*∂*t*}' for the 2 months leading to the peak of the Atlantic Niño (2.1 *e*^−7^K s^−1^) is comparable with the sum of the advection and {*Q*_NET_}' terms (2.0 *e*^−7^K s^−1^). On the other hand, the peak contribution of the zonal {*u*∂*T/*∂*x*}' and meridional {*v*∂*T/*∂*y*}' advection components in driving the ocean mixed layer temperature is delayed until *t*=0, suggesting that the Atlantic Niño event may be essentially triggered by the {*Q*_NET_}' before coupled dynamics sets in (Fig. 7c). Consistent with the results of the thermodynamic configuration of GFDL-CM2.0, the {*Q*_NET_}' is largely controlled by {*Q*_SW_}' and {*Q*_LH_}' (Fig. 7e).

We also conducted heat budget analysis for 500-year integration of the fully coupled configuration of MIROC3.2_medres which exhibits realistic Atlantic Niño seasonality, *σ*{*SST*}' and {*u*}'*|*{*SST*}' coupling and, among the 12 models studied, the ‘best' spatial representation of equatorial Atlantic SST variability. As shown in the right panels (Fig. 7b,d,f), the results of MIROC3.2_medres are basically consistent with those of the GFDL-CM2.0 model. Here again, the missing terms (seen as the residuals in Methods) seem to delay the development of the peak {*SST*}'—nonetheless, the {*Q*_NET_}' terms appear as the driver, and in an integrated sense can explain most of the modelled {∂*T/*∂*t*}'.

## Discussion

An analysis of numerical models of two different levels of ocean-atmosphere coupling suggests a dominant role of stochastic atmospheric forced heat and moisture flux in equatorial Atlantic variability. Recent studies based on *in situ* and satellite observations show that net surface heat flux is important for the development of the climatological-mean cold tongue^33^,^34^,^35^. Thus, it is not entirely surprising that interannual fluctuations of net heat flux could drive cold tongue SST variability.

The Atlantic Niño is typically preceded by changes in the southeast trade winds^1^,^2^,^3^,^4^ associated with perturbations of the St Helena subtropical anticyclone and Benguela Niño evolution^43^,^44^,^45^,^46^. Here we hypothesize that these atmospheric fluctuations can generate the space-time structure and AR(1) spectrum of the equatorial Atlantic Niño via the wind-evaporation-SST feedback mechanisms^20^,^47^, even under motionless ocean conditions. Coupled dynamics can further enhance the characteristic Niño-like structure and energize the interannual variance of the Atlantic Niño, via partly resolving the Bjerknes feedback and changes in mixed layer depth, and in some models this results in an overestimation of the observed variability.

The tropical Atlantic Ocean has experienced profound climate shifts^48^,^49^,^50^ characterized by Niño-like warming trends along the climatological-mean axis of the cold tongue^50^. However, projections of tropical Atlantic climate change, which has implications for the marine ecosystems and socio-economy of the adjacent countries, are uncertain^50^ partly due to major systematic biases of state-of-the-art models in reproducing the basic state^51^,^52^,^53^,^54^. Like the conventional Atlantic Niño, both the warming trends^50^ and model systematic biases are most pronounced during the boreal summer^52^ and exhibit Niño-like spatial structure^51^,^52^,^53^,^54^. The severe biases in mean state can cause unrealistic seasonal cycle in the full-CGCMs, whereas the seasonal cycle in the slab-CGCMs is realistic since it is heavily constrained by the *Q*_flux_ (see Methods), which is constructed using the observed SST. The Atlantic Niño may therefore, in reality, result largely from perturbations of the mean seasonal cycle^55^,^56^. Although we have not considered seasonality explicitly, we do find that a slab-CGCM can simulate the correct structure (not shown). This suggests that further work is required to identify the underlying mechanisms for Atlantic Niño seasonality.

The mean state biases can impact equatorial Atlantic variability, as has been discussed in some recent studies^54^,^57^. However, this relation is non-trivial and there is little evidence for a relation between the mean state biases and variability in our ensemble of coupled models (*R*^2^=0.04). The mean state biases have often been attributed to the atmospheric component of the coupled models; leading hypothesis implicates weakening of the trade winds^51^,^52^. By highlighting the importance of thermodynamic processes, in addition to the more often discussed dynamical feedbacks, our findings may allow for an improved understanding of these biases, and detection and attribution of tropical Atlantic climate change.

Aspects of the thermodynamically driven equatorial Atlantic variability discussed in this study—including its evolution and spatial characteristics—appear similar to the results reported for the Pacific El Niño^21^,^22^,^23^,^24^. Yet, we have shown that thermodynamic processes contribute far less proportion of the overall variance and have less linear control on the state-of-the-art model simulation of the equatorial Pacific event, compared to the Atlantic counterpart. Perhaps, the leading role of atmospheric induced heat and moisture flux explain why the predictability of equatorial Atlantic SST variability, considered a key variable for seasonal climate predictions^4^,^6^, is comparatively low. While the predictability limit of tropical Atlantic SST variability estimated from observations is of the order of 5–8 months, that for the Pacific generally spans >8 months^58^.

If thermodynamic feedbacks constitute the main source of Atlantic Niño variability as described in this study, then potential dynamical predictability may actually be lower than currently thought. Thus, our results call for a re-thinking of the procedures for improving the modelling and predictions of equatorial Atlantic climate that may necessarily be thermodynamically motivated^59^,^60^,^61^, and involving improved representation of atmospheric variability in the coupled models. In this regard, further modelling studies and enhanced observations are needed to confirm the relative importance of dynamical and thermodynamic processes in equatorial Atlantic variability.

## Methods

### Observational SST data sets

The National Oceanic Atmospheric Administration Extended Reconstructed SST version 3b available for 2- × 2- longitude–latitude grids^25^, the Hadley Centre Global Sea Ice and SST^26^ version 1 (1- × 1-) and the Kaplan Extended SST^27^ version 2 (5- × 5-) were analysed. Each data set was linearly de-trended via the least squares method and then the three bi-linearly interpolated onto 1- × 1- longitude–latitude grids to create an ensemble-mean.

### Model selection

Twelve numerical models that contributed skin temperature—a proxy for SST—to the ‘slab ocean control experiment' of the CMIP3 and have integration lengths of ≥30 years were selected for analysis. The slab-CGCM integration lengths (in years) studied are as follows: CCSM3 (51), CGCM3.1_T47 (30), CGCM3.1_T63 (30), CSIRO-Mk3.0 (60), ECHAM5/MPI-OM (100), GFDL-CM2.0 (50), GFDL-CM2.1(100), GISS-ER (120), INM-CM3.0 (60), MIROC3.2_medres(60), MRI-CGCM2.3.2 (150) and UKMO-HadGEM1 (70). Description of these models and experimental designs are available from the CMIP website (http://www-pcmdi.llnl.gov/ipcc/about_ipcc.php).

For each model, the final years of (‘pre-industrial control experiment') integration of the fully coupled configuration (full-CGCM) corresponding to the available length of the slab-CGCM were studied. As in the slab control experiment, greenhouse forcing was held constant in the pre-industrial control experiment and this allows us to explore the intrinsic variability, as the tropical Atlantic Ocean has exhibited marked warming trends^48^,^49^,^50^.

### Thermodynamic coupled model heat budget

In this modelling set-up, the time evolution of the SST is determined by the net heat flux (*Q*_NET_) based on the following thermodynamic equation:


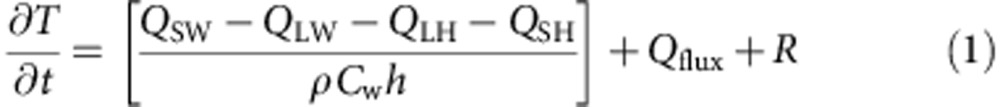


where *ρ*, *h* and *C*_w_ are constants representing the sea water density, ocean mixed layer depth and specific heat capacity of ocean water (*ρ*=10^3^ kg m^−3^; *h*=50 m; *C*_*w*_=4 × 10^3^ J kg^−1^K^*−*1^), respectively, and where *T* is the SST. *Q*_SW_, *Q*_LW_, *Q*_LH_ and *Q*_SH_ denote net downward shortwave radiation at the ocean surface, net upward longwave radiation, surface latent and sensible heat flux, respectively. Note that *Q*_SW_ is positive downward, while the other fluxes are positive upwards. *Q*_flux_ is prescribed monthly climatological-mean ocean heat flux, which contributes to the annual cycle but does not drive SST variability. *R* is a residual term that denotes the sum of boundary flux errors from averages over the Atlantic Niño region and high frequency variability not resolved by the monthly time series analysed. Thus, the variability of ∂*T/*∂*t* is governed by the terms on the right-hand side of the equation enclosed in square bracket [], which denotes the *Q*_NET_.

### Fully coupled model heat budget

Here in addition to the *Q*_NET_ terms, coupling to interactive ocean dynamics denotes the introduction of three-dimensional ocean water advection and thus the exclusion of the restoration term (*Q*_flux_). The temperature tendency is determined as follows:


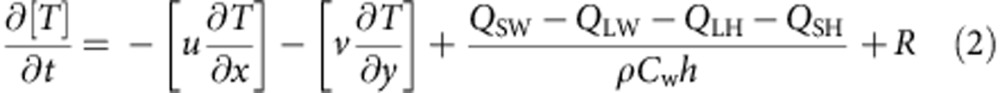


where *u* and *v* are the horizontal ocean current velocities; and where the first two terms on the right-hand side represent zonal and meridional advection terms. *R* is a residual term that represents the sum of boundary flux errors from averages over the Atlantic Niño region, vertical temperature advection [*w*∂*T/*∂z] and unresolved physical processes (for example, diffusion, turbulent mixing and high frequency variability not resolved by the monthly time series analysed). In the fully coupled configuration, the mixed layer depth (*h*) is defined as the depth at which ocean temperatures are 0.5 K lower than those at the surface; thus, *h* changes in space and time. Following the approach of ref. 62, [·] is computed for each variable as the vertical average for the ocean mixed layer:


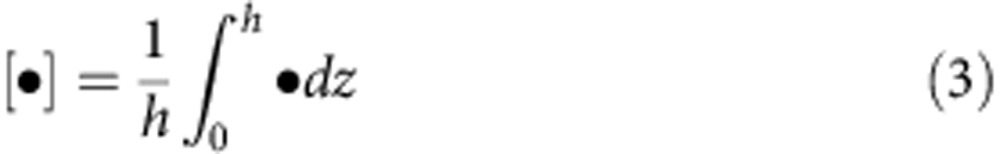


Once the heat budget terms were calculated, each was linearly de-trended, and the climatological-mean annual cycle removed to create an anomaly field {*·*}' via the least squares method and domain-average was then computed for the Atlantic Niño region.

### Mixed layer depth and thermocline

Observed data sets on the climatological-mean ocean mixed layer depth estimated using the temperature criterion described above and thermocline (represented by the depth of the 20 °C isotherm) were taken from the World Ocean Atlas 1994 (WOA94) (ref. 41), available online from: http://www.esrl.noaa.gov/psd/data/gridded/data.nodc.woa94.html. These data sets were analysed alongside those obtained from the European Centre for Medium-range Weather Forecasts Operational Ocean Reanalysis System 3 (ORAS3) (ref. 42) for the period 1959–2009 (http://apdrc.soest.hawaii.edu/datadoc/ecmwf_oras3.php). Modelled mixed layer depth and thermocline were then computed from the historical (‘climate of the twentieth century') experiment of CMIP3 and analysed for the 45-year period from 1955 to 1999.

### Atlantic and pacific Niño indices

The Atlantic Niño index was computed as the domain averaged SST anomaly over the equatorial Atlantic Ocean (3°N–3°S, 0–20W). The Niño-3 index, determined as the domain-average at 5-N–5-S, 90-W–150-W, was used to represent eastern equatorial Pacific El Niño.

### Significance tests

We determined the statistical significance levels based on the two-tailed *P* values using Student's *t-*test, except for the Atlantic Niño spectrum in which significance was estimated as the (lower and upper) 95% confidence bounds of the theoretical AR(1) spectrum using the lag-1 autocorrelation function.

## Additional information

**How to cite this article:** Nnamchi, H. C. *et al.* Thermodynamic controls of the Atlantic Niño. *Nat. Commun.* 6:8895 doi: 10.1038/ncomms9895 (2015).

## Supplementary Material

Supplementary Information Supplementary Figures 1-5.

## References

[b1] Zebiak S. E. Air–sea interaction in the equatorial Atlantic region. J. Clim. 6, 1567–1586 (1993).

[b2] Keenlyside N. S. & Latif M. Understanding equatorial Atlantic interannual variability. J. Clim. 20, 131–142 (2007).

[b3] Carton J. A. *et al.* Decadal and interannual SST variability in the tropical Atlantic Ocean. J. Phys. Oceanogr. 26, 1165–1175 (1996).

[b4] Chang P. *et al.* The cause of the fragile relationship between the Pacific El Niño and the Atlantic El Niño. Nature 443, 324–328 (2006). 16988709 10.1038/nature05053

[b5] Xie S.-P. & Carton J. A. (eds Wang C., Xie S.-P, Carton J. A. AGU Geophysical Monograph 147, 121–142 (2004).

[b6] Giannini A., Saravanan R. & Chang P. Oceanic forcing of Sahel rainfall on interannual to interdecadal time scales. Science 302, 1027–1030 (2003). 14551320 10.1126/science.1089357

[b7] Losada T., Rodríguez-Fonseca B. & Kucharski F. Tropical influence on the summer mediterranean climate. Atmos. Sci. Lett. 13, 36–42 (2012).

[b8] Kucharski F. *et al.* A Gill–Matsuno-type mechanism explains the tropical Atlantic influence on African and Indian monsoon rainfall. Q. J. R. Meteorol. Soc. 135, 569–579 (2009).

[b9] Losada T. *et al.* Tropical response to the equatorial mode: AGCM multimodel approach. Clim. Dyn. 35, 45–52 (2009).

[b10] Rodríguez-Fonseca B. *et al.* Are Atlantic Niños enhancing Pacific ENSO events in recent decades? Geophys. Res. Lett. 36, L20705 (2009).

[b11] Keenlyside N. S. *et al.* Potential of equatorial Atlantic variability to enhance El Niño prediction. Geophys. Res. Lett. 40, 2278–2283 (2013).

[b12] Polo I. *et al.* Processes in the Pacific La Nina onset triggered by the Atlantic Niño. Clim. Dyn. 44, 115–131 (2015).

[b13] Kucharski F. *et al.* Tropical Atlantic influence on Pacific variability and mean state in the twentieth century in observations and CMIP5. Clim. Dyn. 44, 881–896 (2015).

[b14] Bjerknes J. Atmospheric teleconnections from the equatorial Pacific. Mon. Weather Rev. 97, 163–172 (1969).

[b15] Wyrtki K El Niño—The dynamic response of the equatorial Pacific Ocean to atmospheric forcing. J. Phys. Oceanogr. 5, 572–584 (1975).

[b16] Lübbecke J. F. & McPhaden M. J. A comparative stability analysis of Atlantic and Pacific Niño modes. J. Clim. 26, 5965–5980 (2013).

[b17] Richter I. *et al.* Multiple causes of interannual sea surface temperature variability in the equatorial Atlantic ocean. Nat. Geosci. 6, 43–47 (2013).

[b18] Brandt P. *et al.* Interannual atmospheric variability forced by the deep equatorial Atlantic ocean. Nature 473, 497–500 (2011). 21593764 10.1038/nature10013

[b19] Meehl G. A. *et al.* The WCRP CMIP3 multimodel dataset: a new era in climate change research. Bull. Amer. Meteorol. Soc. 88, 1383–1394 (2007).

[b20] Xie S.-P. & Philander S. G. H. A coupled ocean-atmosphere model of relevance to the ITCZ in the eastern Pacific. Tellus A 46, 340–350 (1994).

[b21] Dommenget D. The slab ocean El Niño. Geophys. Res. Lett. 37, L20701 (2010).

[b22] Clement A. *et al.* Rethinking the ocean's role in the southern oscillation. J. Clim. 24, 4056–4072 (2011).

[b23] Zhang H., Clement A. & Di Nezio P. The South Pacific meridional mode: a mechanism for ENSO-like variability. J. Clim. 27, 769–783 (2013).

[b24] Dommenget D. *et al.* Analysis of the slab ocean El Niño atmospheric feedbacks in observed and simulated ENSO dynamics. Clim. Dyn. 42, 3187–3205 (2014).

[b25] Smith T. M. *et al.* Improvements to NOAA's historical merged land–ocean surface temperature analysis (1880–2006). J. Clim. 21, 2283–2296 (2008).

[b26] Rayner N. A. *et al.* Global analyses of sea surface temperature, sea ice, and night marine air temperature since the late nineteenth century. J. Geophys. Res. 108, 4407 (2003).

[b27] Kaplan A. *et al.* Analyses of global sea surface temperature 1856-1991. J. Geophys. Res. 103, 567–589 (1998).

[b28] Taylor K. E. Summarizing multiple aspects of model performance in a single diagram. J. Geophys. Res. 106, 7183–7192 (2001).

[b29] Latif M. & Grötzner A. The equatorial Atlantic oscillation and its response to ENSO. Clim. Dyn. 16, 213–218 (2000).

[b30] Hasselmann K. Stochastic climate models Part I. Theory. Tellus 28, 473–485 (1976).

[b31] Frankignoul C. & Hasselmann K. Stochastic climate models. Part 2. Application to sea-surface temperature variability and thermocline variability. Tellus 29, 284–305 (1977).

[b32] Delworth T. L. *et al.* GFDL's CM2 global coupled climate models. Part I: Formulation and simulation characteristics. J. Clim. 19, 643–674 (2006).

[b33] Wade M., Caniaux G. & du Penhoat Y. Variability of the mixed layer heat budget in the eastern equatorial Atlantic during 2005–2007 as inferred using Argo floats. J. Geophys. Res. 116, C08006 (2011).

[b34] Foltz G. R. *et al.* Seasonal cycle of the mixed layer heat budget in the northeastern tropical Atlantic Ocean. J. Clim. 26, 8169–8188 (2013).

[b35] Hummels R. *et al.* Diapycnal heat flux and mixed layer heat budget within the Atlantic cold tongue. Clim. Dyn. 43, 3179–3199 (2014).

[b36] Trzaska S. *et al.* South Atlantic variability arising from air–sea coupling: local mechanisms and tropical–subtropical interactions. J. Clim. 20, 3345–3365 (2007).

[b37] Hu Z. & Huang B. Physical processes associated with the tropical Atlantic SST meridional gradient. J. Clim. 19, 5500–5518 (2006).

[b38] Hu Z. & Huang B. Physical processes associated with the tropical Atlantic SST gradient during the anomalous evolution in the southeastern ocean. J. Clim. 20, 3366–3378 (2007).

[b39] Ding H., Keenlyside N. S. & Latif M. Equatorial Atlantic interannual variability: role of heat content. J. Geophys. Res. 115, C09020 (2010).

[b40] Chang P. *et al.* Climate fluctuations of tropical coupled systems—The role of ocean dyynamics. J. Clim. 19, 5122–5174 (2006).

[b41] Monterey G. I. & Levitus S. *Climatological cycle of mixed layer depth in the world ocean* pp 5 (U.S. Gov. Printing Office, Washington, D.C., 1997).

[b42] Balmaseda M. A. *et al.* The ECMWF ocean analysis system: ORA-S3. Mon. Weather Rev. 136, 3018–3034 (2008).

[b43] Lübbecke J. F. *et al.* On the connection between Benguela and equatorial Atlantic Niños and the role of the South Atlantic Anticyclone. J. Geophys. Res. 115, C09015 (2010).

[b44] Richter I. *et al.* On the triggering of Benguela Niños: Remote equatorial versus local influences. Geophys. Res. Lett. 37, L20604 (2010).

[b45] Nnamchi H. C., Li J. P. & Anyadike R. N. C. Does a dipole mode really exist in the South Atlantic Ocean? J. Geophys. Res. 116, D15104 (2011).

[b46] Lübbecke J. F. *et al.* Variability in the South Atlantic anticyclone and the Atlantic Niño mode. J. Clim. 27, 8135–8150 (2014).

[b47] Chang P., Ji L. & Li H. A decadal climate variation in the tropical Atlantic Ocean from thermodynamic air-sea interactions. Nature 385, 516–518 (1997).

[b48] Deser C. *et al.* Twentieth century tropical sea surface temperature trends revisited. Geophys. Res. Lett. 37, L10701 (2010).

[b49] Xie S.-P. *et al.* Global warming pattern formation: Sea surface temperature and rainfall. J. Clim 23, 966–986 (2010).

[b50] Tokinaga H. & Xie S.-P. Weakening of the equatorial Atlantic cold tongue over the past six decades. Nat. Geosci. 4, 222–226 (2011).

[b51] Richter I. & Xie S.-P. On the origin of equatorial Atlantic biases in coupled general circulation models. Clim. Dyn. 31, 587–598 (2008).

[b52] Grodsky S. A. *et al.* Tropical Atlantic Biases in CCSM4. J. Clim. 25, 3684–3701 (2012).

[b53] Richter I. *et al.* Equatorial Atlantic variability and its relation to mean state biases in CMIP5. Clim. Dyn 42, 171–188 (2014).

[b54] Ding H. *et al.* The impact of mean state errors on equatorial Atlantic interannualvariability in a climate model. J. Geophys. Res. 120, 1133–1151 (2015).

[b55] Burls N. J., Reason C. J. C., Penven P. & Philander S. G. Similarities between the tropical Atlantic seasonal cycle and ENSO: an energetics perspective. J. Geophys. Res. 116, C11010, doi:10.1029/2011JC007164 (2011).

[b56] Burls N. J. *et al.* Energetics of the tropical Atlantic zonal mode. J. Clim. 25, 7442–7466 (2012).

[b57] Munoz E. *et al.* Mean and variability of the tropical Atlantic Ocean in the CCSM4. J. Clim. 25, 4860–4882 (2012).

[b58] Li J. P. & Ding R. Q. Temporal–spatial distribution of the predictability limit of monthly sea surface temperature in the global oceans. Int. J. Climatol. 33, 1936–1947 (2013).

[b59] Chang P. *et al.* Tropical Atlantic seasonal predictability: the roles of El Niño remote influence and thermodynamic air-sea feedback. Geophys. Res. Lett. 30, 1501 (2003).

[b60] Barreiro M. & Chang P. A linear tendency correction technique for improving seasonal prediction of SST. Geophys. Res. Lett. 31, L23209 (2004).

[b61] Batté L. & Déqué M. A stochastic method for improving seasonal predictions. Geophys. Res. Lett. 39, L09707 (2012).

[b62] Li T. *et al.* Relative role of dynamic and thermodynamic processes in the development of the Indian Ocean dipole: an OGCM diagnosis. Geophys. Res. Lett. 29, 2110 (2002).

