# Supplementary material for: Thermodynamic controls of the Atlantic Niño
Source: Nat Commun. 2015 Nov 26;6:8895. doi: 10.1038/ncomms9895 (PMC4674767; doi:10.1038/ncomms9895)
Supplement: Supplementary Information — Supplementary Figures 1-5. [file ncomms9895-s1.pdf]

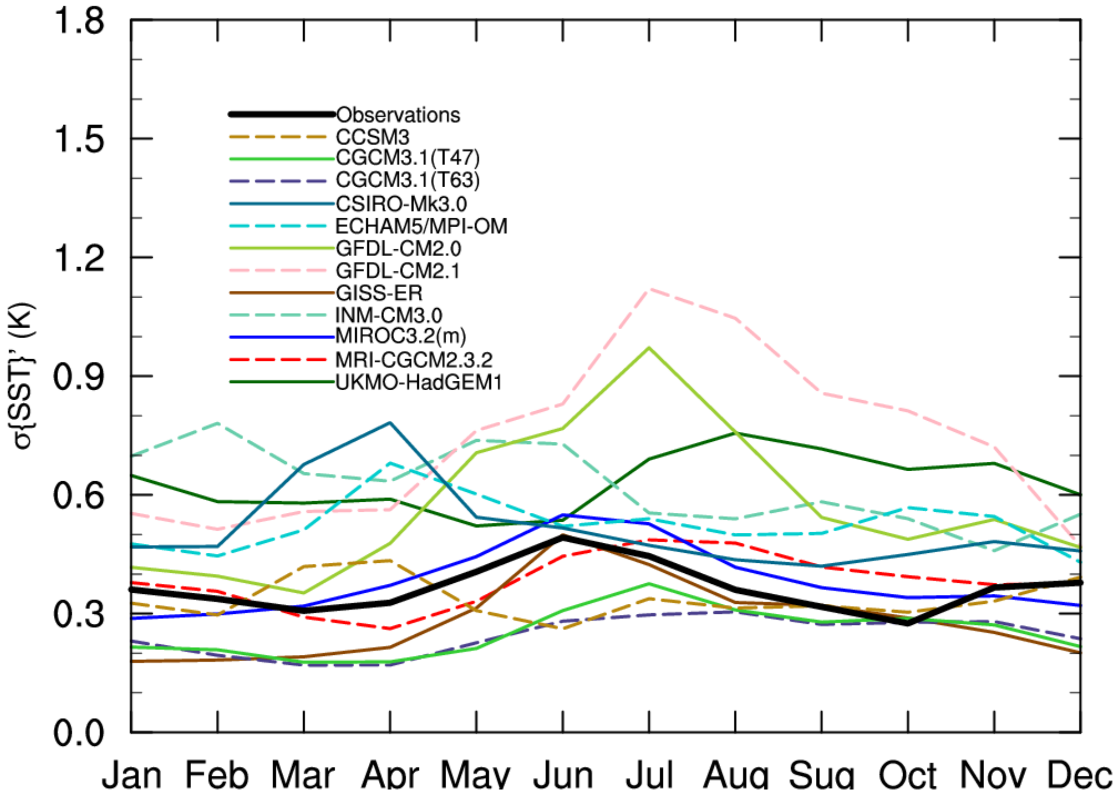

Supplementary Figure 1| Seasonality of the Atlantic Niño. Curves show the monthly standard deviation of the Atlantic Niño SST index from observations and the fully coupled configuration of the CMIP3 models.

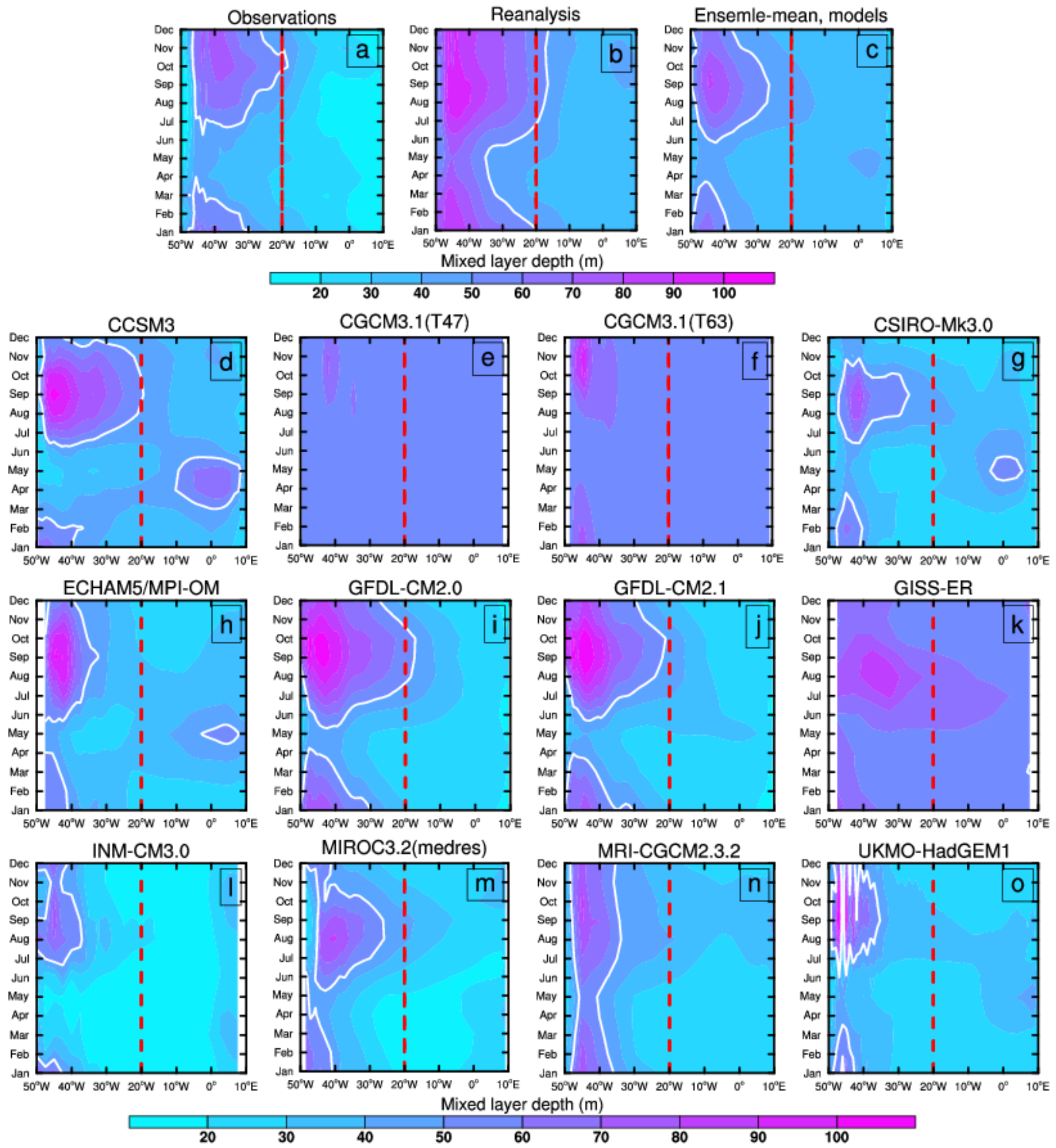

Supplementary Figure 2| Equatorial Atlantic ocean mixed layer depth. Shown are the seasonal cycle of the meridional (3°S-3°N) average of the ocean mixed layer depth across the equatorial Atlantic. **a,** Word Ocean Atlas 94. **b,** ORAS3. **c,** Ensemble-mean of the 12 CMIP models. **d-o**, The individual CMIP3 models. White contour delineates regions where the mixed layer depth exceed 50 m, assumed in the Slab-CGCMs. The depths exceed 50 m throughout the region in models without white curves (CGCM3.1_T47,CGCM3.1_T63 and GISS-ER). In each panel, dashed red line (along 20°W) delineates the eastern from western equatorial Atlantic Ocean.

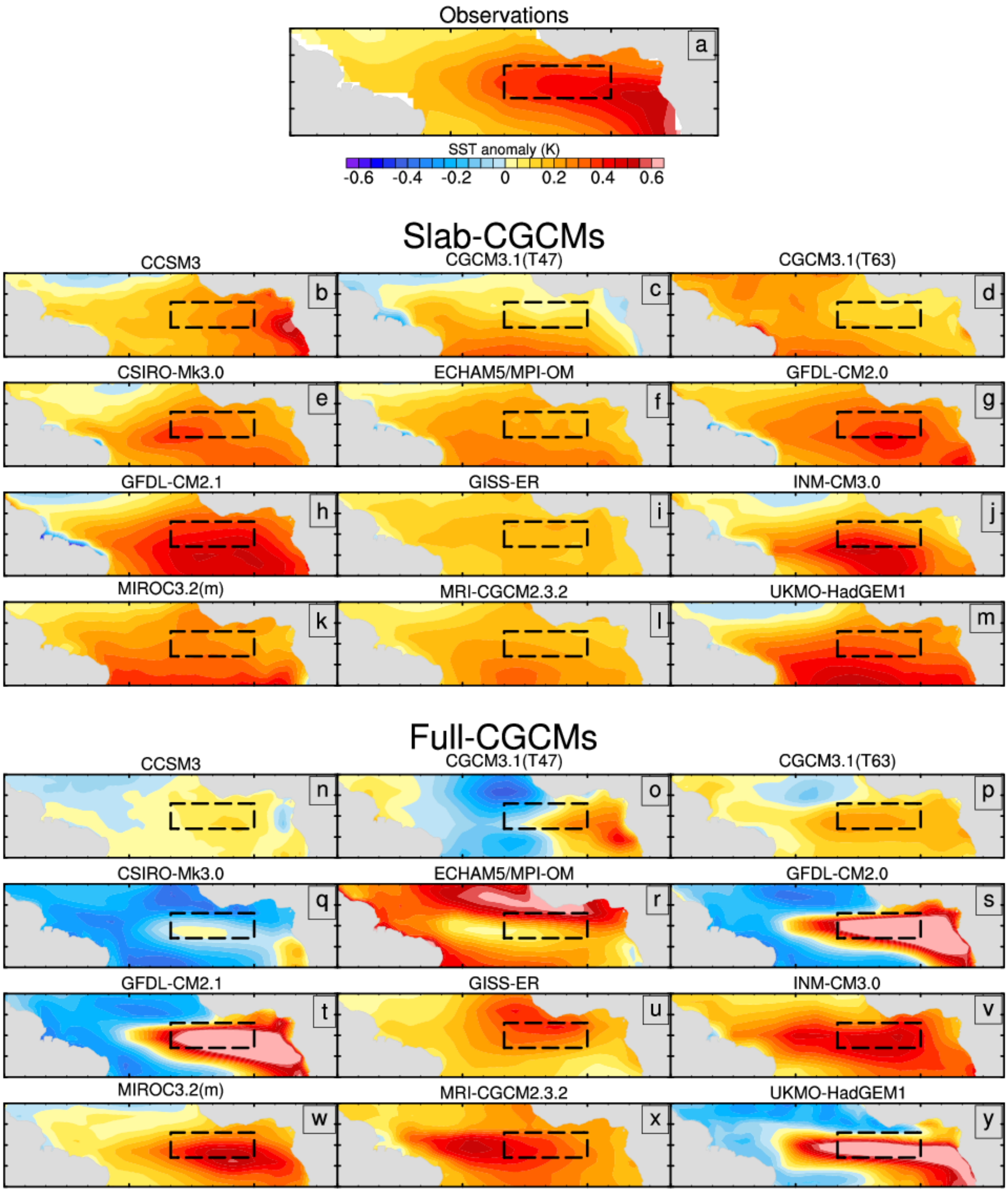

Supplementary Figure 3| Leading empirical orthogonal functions (EOF) of SST anomalies over equatorial Atlantic Ocean. The leading EOF mode of SST for JJA was computed over the region (10°N-10°S, 20°E-60°W) and the spatial pattern determined by regressing SST anomalies onto the corresponding EOF time series. **a,**T Observed EOF pattern based for 1984-2013. **b-m,** EOF patterns simulated by the Slab-CGCMs. **n-y,** EOF patterns simulated by the Full-CGCMs. The model names are indicated on the top of each panel.

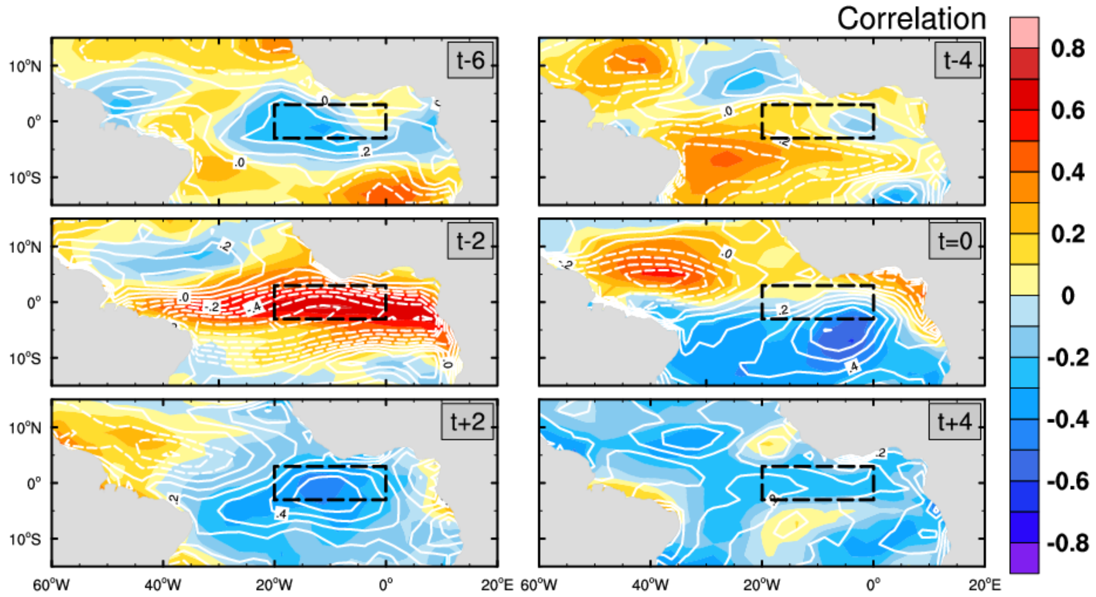

Supplementary Figure 4| Evolution of latent and net heat fluxes associated with the Atlantic Niño in thermodynamic configuration of GFDL-CM2.0. Correlation maps of the monthly anomalies of surface heat flux (colour scale) and latent heat flux(contours) and the Atlantic Niño index fixed at *t=0*, corresponding to July.

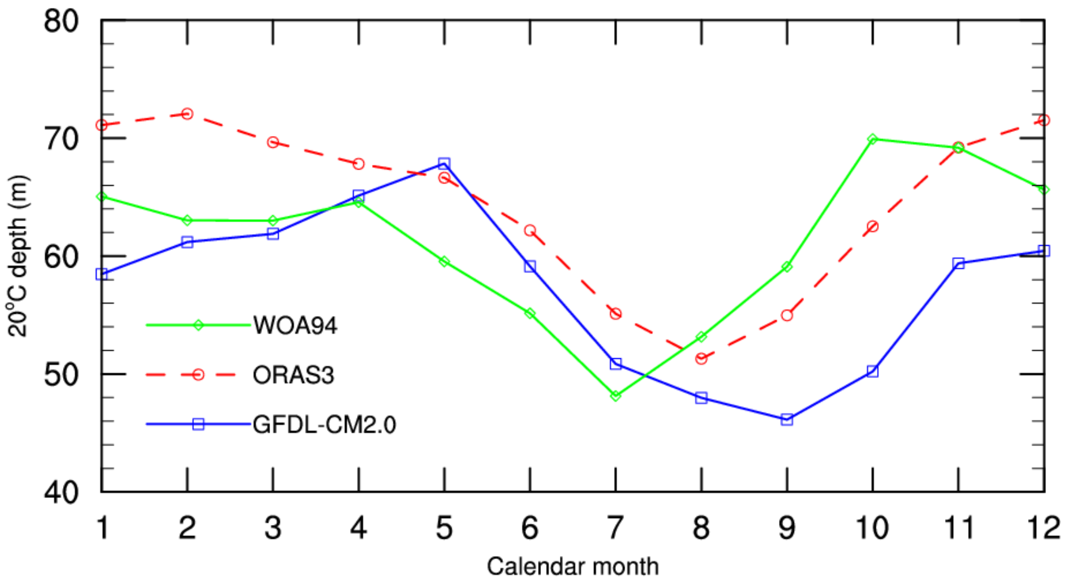

Supplementary Figure 5| Equatorial Atlantic thermocline depth. Shown are the annual cycle of the depth of 20°C isotherm averaged in the Atlantic Niño region. The 20°C isotherm depth are computed from GFDL-CM2.0 (blue), ECMWF Ocean Reanalysis, System 3 and World Ocean Atlas 94 (green).
